# Deciphering the role of Nuclear Factor-κB in cellular senescence

**DOI:** 10.18632/aging.100390

**Published:** 2011-09-26

**Authors:** Simon Vaughan, Parmjit S. Jat

**Affiliations:** Department of Neurodegenerative Disease, UCL Institute of Neurology, Queen Square, London WC1N 3BG, UK

**Keywords:** cellular senescence, ageing, NF-κB, SASP

## Abstract

Cellular senescence is a program of irreversible cell cycle arrest that cells undergo in response to a variety of intrinsic and extrinsic stimuli including progressive shortening of telomeres, changes in telomeric structure or other forms of genotoxic and non-genotoxic stress. The role of nuclear factor-κB in cellular senescence is controversial, as it has been associated with both proliferation and tumour progression, and also with growth arrest and ageing. This research perspective focuses on the evidence for a functional relationship between NF-κB and senescence, and how disruption of the NF-κB pathway can lead to its bypass.

Somatic cells usually undergo a limited number of divisions termed the Hayflick limit [1]. When this limit is reached, cellular senescence, a permanent cell cycle arrest is triggered in conjunction with characteristic changes in cell morphology and physiology. Both endogenous and exogenous cues such as telomere attrition, or stress in the form of oncogene activation or DNA damage can induce senescence by activating the tumour suppressor p53 [2-4]. Activation of p53 increases levels of p21^CIP1^, which inhibits cyclinE-CDK2 complexes from phosphorylating the pRB family of proteins. This leads to activation of the pRB tumour suppressor pathway in which the hypophosphorylated pRB family of proteins complex with E2F heterodimers, thereby preventing cell cycle progression [2-4]. P21^CIP1^ can also arrest cell cycle progression by inhibiting cyclinA/B-CDK1 and cyclinD/CDK4 complexes [5]. The pRB pathway can also be activated independently of p53 by upregulation of p16^INK4A^ upon non-genotoxic stress, which inhibits cyclinD-CDK4/6 complexes from phosphorylating the pRB family of proteins [2-4].

Senescence can dampen the repair and regenerative ability of a tissue due to an increased population of growth arrested cells and a diminished stem cell population; and thereby play a hand in tissue ageing. It can also act as a tumour suppressor mechanism by preventing the proliferation of potentially cancerous cells. Bypass of the cell cycle arrest can lead to a limitless replicative potential, a key step in malignant transformation [6]. Blagosklonny has proposed that senescence involves two steps: cell cycle arrest and an activation of intracellular signalling pathways which result in cellular hypertrophy and a pro-inflammatory and hyper-secretory phenotype [7-9]. Although the mechanisms underlying the cell cycle arrest are broadly known, there remain questions as to what makes the growth arrest irreversible, the identity of the critical down-stream targets and how the growth arrest is fine tuned. It also remains to be determined as to what the other intracellular signal transduction pathways involved are and how the diverse signals that result in senescence are integrated.

The role of nuclear factor (NF)-κB in senescence is controversial, as it is divisively associated with proliferation and tumour progression, and contrastingly with growth arrest and ageing. The NF-κB family of transcription factors are ubiquitously expressed and regulate the response to cellular and environmental stress [10]. In the canonical pathway (Figure 1), homo- and heterodimers of these proteins associate with a cytosolic inhibitor, inhibitor of NF-κB (IκB), resulting in their retention in the cytoplasm. Phosphorylation of IκB by the IκB kinase (IKK) complex (IKKα, IKKβ and IKKγ/NEMO) and its subsequent degradation by the 26S proteasome unmasks a nuclear localisation signal on NF-κB, leading to their nuclear translocation and induction of target gene transcription [10-14]. The canonical pathway is activated by a wide array of receptors including those for inflammatory cytokines, pathogen-associated molecules and antigen receptors and results in the activation of IKKβ [11].

**Figure 1:**
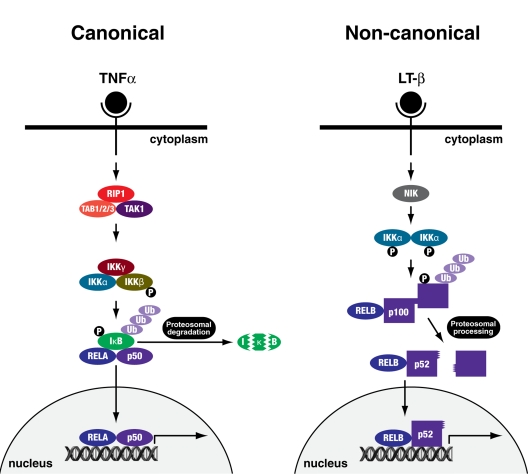
Canonical and non-canonical NFκB signalling pathways The canonical pathway can be activated by cytokines, or molecules of bacterial or viral origin such as TNFα. IKKγ/NEMO and IKKβ are essential for the phosphorylation and subsequent ubiquitination and proteosomal degradation of IκB, leading to nuclear translocation of the RELA-p50 transcription factor complex. The non-canonical pathway is activated by a more specific set of stimuli such as lymphotoxin β (LT-β). NIK and IKKα are essential for phosphorylation and subsequent ubiquitination and proteosomal processing of p100 giving rise to a transcriptionally active RELB-p52 complex.

The non-canonical pathway (Figure 1) depends on the processing of the p100 precursor protein to produce mature p52 (NF-κB2) protein. This involves the NF-κB inducing kinase (NIK) phosphorylating and activating IKKα, which in turn phosphorylates p100, marking it for ubiquitination and proteasomal processing [11]. The transactivation potential of NF-κB complexes can be further potentiated by direct phosphorylation of RELA, for example, by GSK-3β, RSK1 or p38MAPK [15, 16]. How, though, is NF-κB signalling activated in senescence?

Persistent inflammation is an important underlying condition that aids tumour development and is characterised by the activation of NF-κB signalling. The NF-κB family of proteins have been implicated in tumour development due to their sequence similarity to the viral oncogene v-Rel [17, 18]. Mutations that lead to an increase in NF-κB activity have been observed in several cancers [17-20], though NF-κB is not believed to be required for tumour initiation; it is thought to play a role in tumour promotion [20]. NF-κB regulates apoptosis genes, with a block in NF-κB leading to an increase in apoptosis, suggesting that NF-κB can prevent the death of cancer cells [20]. TBK1, a non-canonical IKK, has been shown to be crucial in the context of oncogenic KRAS, by activating anti-apoptotic signals via NF-κB to aid the survival of cancer cells, thus promoting tumour development [21]. In addition to the inhibition of apoptosis, NF-κB may stimulate other cancer-associated phenotypes [17, 18]. In melanoma, NF-κB (RELA-p50) is commonly found to be overactive, despite the absence of any reported mutations. It can apparently be activated by oncogenic BRAF, autocrine cytokines such as TNF, hypoxia via HIF1α and AKT (protein kinase B) or oncogenic NRAS via AKT. NF-κB has been implicated in invasiveness as well as survival, inducing the “epithelial-mesenchymal transition” via TWIST1 and SNAI1 [22]. It has also been argued that deletion of IKKβ can induce an anti-tumourigenic phenotype [23], though this may be independent of NF-κB as IKKs can also function separately of NF-κB signalling. Moreover constitutively active IKKβ has been shown to delay Ha-RasV12 oncogene-induced senescence (OIS) in normal primary human fibroblasts by suppressing the DNA damage response (DDR), demonstrating that NF-κB activity may help to overcome the growth-suppressive effects of OIS [24].

In parallel to its role in tumour development, chronic inflammation also plays an important role in the ageing process. Increased expression of inflammatory markers and an associated increase in NF-κB DNA binding activity have been demonstrated in cells from older donors over cells from younger donors [25]. This may be partly due to the constitutive activation of NF-κB as a result of accumulated oxidative stress during ageing, which results in persistent inflammation [26]. Adaptive immunity also wanes with age placing an extra demand on innate immunity, thereby contributing to the constitutive activation of NF-κB and chronic inflammation [27]. NF-κB has been shown to be strongly associated with progeria, a disease of premature ageing [28]. Aged tissues exhibit SA β-gal activity and enhanced p16^INK4A^ expression, both of which are reduced upon NF-κB inhibition [28]. Moreover inhibition of NF-κB in old tissue was shown to result in expression of genes characteristic of younger tissue [28].

Recently, Rovillain et al. [29] used a novel system of conditionally immortal human fibroblasts, which can be induced to undergo senescence to show that senescence was associated with activation of NF-κB signalling and its perturbation by silencing of NF-κB subunits, or by the presence of the super repressor of NF-κB (IκB-SR), led to the evasion of senescence. They further showed that senescent cells were enriched for the transcriptionally active form of RELA, with phosphorylation at serine 536 [29]. At the same time, Campisi and colleagues showed that increased NF-κB transcriptional activity due to p38MAPK activation was sufficient to induce the senescence associated secretory phenotype [SASP] of senescent fibroblasts [30]. Subsequently, Bertolotto and colleagues [31] have shown that a poly (ADP-ribose) polymerase-1 (PARP) /NF-κB signalling cascade can induce senescence and SASP in melanoma cells.

Although it is not known how NF-κB signalling is activated in senescence, a pathway linking genotoxic stress to activation of NF-κB (Figure 2) in mouse embryo fibroblasts has recently been elucidated by Scheidereit and colleagues [32, 33]. They have shown that genotoxic stress activates PARP-1 and ataxia telangiectasia mutated kinase (ATM) giving rise to synthesis of poly ADP-ribose leading to the assembly of ATM and an IKKγ, PIASy complex. This nucleo-plasmic signalosome triggers PIASy-mediated IKKγ sumoylation and IKKγ phosphorylation by ATM. The modified IKKγ is then translocated to the cytoplasm and integrates into the IKK complex. The activated ATM is also translocated into the cytoplasm, leads to the activation of TRAF6 and induces a TRAF6-Ubc13-mediated polyubiquitin-dependent cascade, resulting in TAB2-dependent phosphorylation of TAK1. Thus both PARP-1 and ATM signalling converge to catalyse monoubiquitination of IKKγ which is required for phosphorylation of IKKγ by TAK1 and enzymatic activation of IKK. It remains to be determined if these pathways are active in senescence. However ATM has been shown to promote senescence by activating p53 and suppression of its activity leads to bypass of senescence [34, 35].

**Figure 2:**
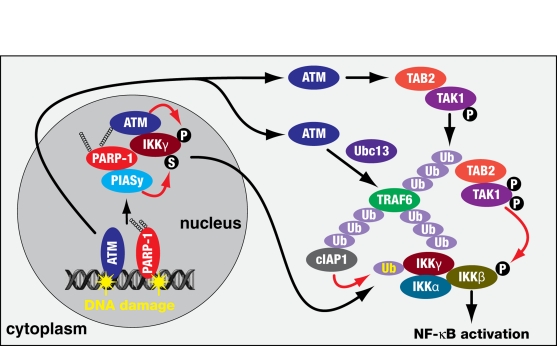
A pathway for activation of NF-κB signalling by genotoxic stress (adapted from Hinz et al. [33]) DNA damage recruits and activates PARP-1 and ATM, with PARP-1 producing poly(ADP-ribose) leading to assembly of IKKγ/NEMO and PIASy and ATM. PIASy and ATM SUMOylate and phosphorylate IKKγ/NEMO respectively which is then translocated to the cytoplasm to form the IKK complex. ATM independently translocates to the cytoplasm to induce TRAF6-Ubc13 polyubiquitination, which promotes cIAP dependent IKKγ/NEMO ubiquitination which then promotes TAB2-TAK1 dependent phosphorylation and activation of IKKβ.

Insulin/IGF1 signalling can promote NF-κB signalling and potentiate ageing and age-related degeneration by activation of the phosphoinositide 3-kinase (PI-3K) /AKT pathway (Figure 3) [16]. Although a role for the insulin/IGF signalling in human ageing remains controversial, this pathway has been conserved in evolution from metazoans to mammals and acts via PI-3K/AKT signalling to maintain cellular homeostasis. It is in this pathway that the functions of FOXO3a and SIRT1 (the human homologue of yeast Sir2) intersect with NF-κB signalling. FOXO3a is a transcription factor that can extend life span and dampen ageing [36]. Activation of insulin/IGF1 signalling results in phosphorylation of FOXO proteins at a number of sites leading to their retention in the cytoplasm [16]. Caloric restriction (CR) reduces signalling, leading to FOXOs remaining unphosphorylated and being translocated to the nucleus to enhance the expression of longevity genes [37]. AKT phosphorylates FOXO3a, but IKKβ can also perform this role [38]. AKT can stimulate NF-κB signalling by activating the IKK complex by phosphorylating IKKβ [16]. AKT itself can be activated by loss of the tumour suppressor phosphatase and tensin homologue, PTEN, which is one of the most frequently altered tumour suppressors in cancer particularly prostate cancer [39]. PTEN converts the membrane lipid second messenger PIP3 to PIP2, thereby inactivating downstream signalling. Pandolfi and colleagues found that loss of PTEN in mice induces senescence and that this PTEN-loss-induced-senescence is distinct from OIS [40].

**Figure 3:**
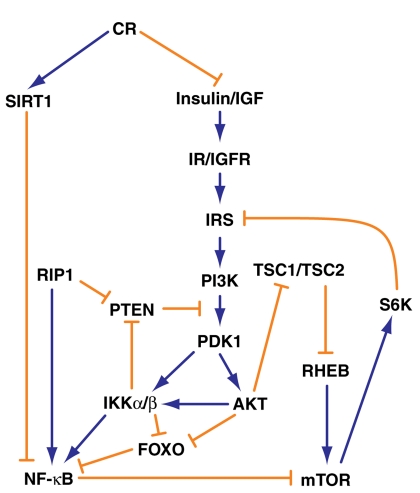
A schematic illustration of the pathways linking caloric restriction, Insulin/IGF, PI-3K/AKT and NF-κB signalling (adapted from Salminen and Kaarniranta [16] and Chauncey et al. [48]) Sustained insulin/IGF signalling promotes NF-κB signalling by activation of PI-3K/AKT pathway whereas caloric restriction (CR) reduces NF-κB signalling by dampening PI-3K/AKT and increasing expression of SIRT1. The PI3K/AKT and NF-κB pathways are also reportedly linked via RIP1.

CR may also increase expression of SIRT1 (Figure 3), a nicotinamide adenosine dinucleotide (NAD) dependent deacetylase which binds and deacetylates p65/RELA, undoing its acetylation by histone acetyl transferases (HATs) and suppressing its transcriptional activity [41]. SIRT1 has been found to protect mice from metabolic damage and ageing associated diseases such as metabolic syndrome, Alzheimer's disease and some types of cancer [42, 43]. Interestingly, Rovillain et al. found that in the conditionally immortal human fibroblasts, SIRT1 was down-regulated and TMEM9B was up-regulated upon senescence and the differential expression was reversed when senescence was bypassed [29, 44]. TMEM9B, is a glycosylated lysosomal transmembrane protein that can stimulate NF-κB activity and acts downstream of receptor interacting protein 1 (RIP1) and upstream of MAPK and IκB kinases at the level of TAK1 [45, 46]. RIP1 has been reported to be the link between the PI-3K/AKT/mTOR and NF-κB pathways. It activates signalling downstream of PI-3K/AKT by down-regulating PTEN and interrupting the negative feedback loop that extends from mTOR to PI-3K/AKT by inhibiting mTOR via NF-κB [47, 48].

FOXM1 is a highly conserved forkhead transcription factor that regulates the expression of numerous regulators of cell cycle progression, particularly in the G2 phase [49]. FOXM1 is down-regulated upon replicative senescence [50] and also markedly reduced in cells from elderly patients as well as those with progeria [51]. Marcu and colleagues have shown that acute activation of NF-κB in mouse embryo fibroblasts leads to growth arrest in association with repression of FoxM1 and cell cycle regulated genes that are known targets of E2F or FOXM1 [52]. Rovillain et al. [29] found that FOXM1 was downregulated upon senescence in the conditionally immortal fibroblasts and that a constitutively active FOXM1 bypasses senescence, suggesting that activation of NF-κB activity may induce senescence by repressing FOXM1.

Senescent cells secrete growth factors, proteases, and inflammatory cytokines as part of the SASP [53-60]. Despite the ability of these factors to promote a malignant phenotype in a paracrine manner, SASP components can bolster senescence in an autocrine manner [53-60], and some even promote the clearance of senescent cells by the immune system [61]. SASP can thus be stimulatory or inhibitory for tumourogenesis depending on the biological context, with it possibly promoting tumourigenesis in aged tissues [30, 59, 60]. The stress-activated kinase p38 has been shown to regulate SASP; inhibition supresses SASP secretion, whereas constitutive activation is sufficient for induction. The effect of p38MAPK on SASP is mediated by increased transcriptional activity of NF-κB [30]; p38MAPK has also been suggested to inhibit FoxM1 expression [62].

In this perspective, we have reviewed the recent findings about the role of NF-κB signalling in ageing and senescence, and weighed up the contradictory evidence. Previous studies had proposed NF-κB signalling as a proliferative message, as well as others conversely implicating NF-κB signalling in growth arrest. With such controversy, many questions remain to be answered. Our latest results show that silencing of NF-κB allowed cells to bypass senescence, and that activation of NF-κB signalling leads to upregulation of genes associated with SASP [29] and suppression of FOXM1 and other cell cycle progression genes. Based on these findings and others discussed here, we propose that activation of NF-κB signalling, in normal somatic cells, promotes senescence and by extension acts to enhance ageing. A better understanding of the role of NF-κB signalling and how it gets activated will increase our knowledge of the pathobiology of ageing and age related diseases.
